# Transcriptomic characterization of mucosal hemocytes in the eastern oyster (*Crassostrea virginica*) underlines their potential role as immune sentinels

**DOI:** 10.3389/fimmu.2026.1773651

**Published:** 2026-03-30

**Authors:** Sherif Abdallah, Denis Grouzdev, Emmanuelle Pales Espinosa, Bassem Allam

**Affiliations:** Marine Animal Disease Laboratory, School of Marine and Atmospheric Sciences, Stony Brook University, Stony Brook, NY, United States

**Keywords:** agranulocytes, bivalves, granulocytes, hemocytes, mucosal immunity, oyster, RNA-seq

## Abstract

Substantial progress has been made in understanding hemocytes within the hemolymph (herein referred to as circulatory hemocytes), which are essential for immune defense and various physiological functions in bivalve mollusks. Yet, our knowledge of peripheral immunity, particularly the role of hemocytes associated with mucosal surfaces covering pallial (gills, mantle) organs (herein referred to as mucosal hemocytes), remains limited. While mucosal and circulatory hemocytes share similar morphologies, they exhibit distinct functional profiles and cell surface epitopes. The molecular mechanisms underlying these functional differences remain poorly understood. To address this knowledge gap, we characterized gene expression profiles (using RNA sequencing) in granulocytes and agranulocytes isolated via flow cytometry from hemolymph and mucus covering pallial tissues of the eastern oyster, *Crassostrea virginica*. Mucosal hemocytes showed overexpression of genes related to cell motility, cytokine activity, signaling, and cell adhesion, supporting the hypothesis that they may have sentinel functions. Despite a consistent dichotomy in gene expression between granulocytes and agranulocytes across both body fluids, it was more pronounced in circulatory hemocytes. Circulatory granulocytes showed functions linked to phagocytosis and pathogen killing, whereas circulatory agranulocytes overexpressed genes associated with mitosis and early inflammation compared to their mucosal counterparts. To our knowledge, this is the first study combining flow cytometry sorting and transcriptomic methods to characterize hemocytes from different body fluids in a marine invertebrate. Results underline the potential role of mucosal hemocytes as immune sentinels, although more studies, possibly using single-cell transcriptomic methods and functional assays associated with pathogen challenge experiments, are needed to probe their specific functions.

## 1 Introduction

Marine bivalves possess an open circulatory system and rely on innate immunity, driven by both humoral and cellular responses (1). Humoral defense factors in their plasma include lysosomal enzymes, soluble lectins, antimicrobial proteins/peptides, and various protease inhibitors (2–5). Cellular immune responses in marine bivalves depend on hemocytes, which are essential for pathogen phagocytosis, encapsulation, and the release of cytotoxic molecules to destroy and eliminate pathogens (6–9). In addition, hemocytes also play a role in shell biomineralization, reproduction, transport of nutrients, food digestion, and excretion (10–13).

Hemocytes have been classified based on their morphological characteristics, biological functions, physicochemical properties, and ultrastructural features. Techniques such as immunostaining of cell surface proteins (14, 15), flow cytometry (16, 17), and density-gradient centrifugation (18) have been employed to characterize hemocyte populations. It is generally accepted that hemocytes comprise two main subtypes; granulocytes and agranulocytes (5, 19–22). Granulocytes contain large, dense cytoplasmic granules rich in peroxidase, phosphatase, and melanin (23, 24), while agranulocytes are distinguished by their hyaline cytoplasm and smooth appearance (25, 26). It should be noted, however, that some recent studies have reported a broader diversity of hemocytes in bivalves with no less than seven different sub-types proposed in the Pacific oyster (27, 28).

Granulocytes are highly mobile and more effective in phagocytosis and pathogen neutralization than agranulocytes, largely due to their elevated production of reactive oxygen species and hydrolytic enzymes that facilitate intracellular pathogen killing (7, 29–31). Meanwhile, agranulocytes play a key role in early inflammatory responses, with greater capacity for wound infiltration and encapsulation of foreign particles (7, 27, 32, 33). However, little is known about the molecular mechanisms that regulate the functional differentiation between granulocytes and agranulocytes.

Mucosal epithelia are the primary interface for animal-microbe interactions (34–36) and are a crucial component of both innate and acquired immunity (37). In vertebrates, dendritic cells and neutrophils play a central role in detecting and responding to microbes at these mucosal interfaces (38, 39). These sentinel cells actively migrate through epithelial layers to monitor the microbial composition of mucosal tissues (e.g., gut, respiratory tract) before returning to deeper tissues to activate the systemic immune system, such as through cytokine release (40, 41). In anamniote vertebrates and invertebrates, much research has explored host-microbe interactions after infection has been established (42–46), yet the dynamics of microbial encounters at mucosal interfaces during initial contact remain poorly understood. In particular, the roles and characteristics of the mobile cellular components of mucosal immunity in these animals are still not well defined.

In marine bivalves, pallial organs, such as mantle, palps, and gills are well irrigated with hemolymph to facilitate nutrient exchange and oxygen extraction from the surrounding environment. Yonge (47) and Takatsuki (48) first documented hemocytes in the mucus (herein designated as mucosal hemocytes) covering the gut lumen and pallial organs, respectively. More recently, our research also identified the presence of hemocytes in mucus covering the mantle and gills of oysters (49–51) and clams (52, 53), confirming Takatsuki’s observations. Notably, oyster mucosal hemocytes have been observed to translocate from mucosal surfaces to the circulatory system within hours (9, 49), suggesting that mucosal hemocytes may function similarly to dendritic cells in invertebrates.

This study was designed to generate a comparative transcriptomic analysis that explores the molecular differences between granulocytes and agranulocytes across the hemolymph and pallial mucus. Hemocyte subpopulations of the eastern oyster, *C. virginica*, were sorted using flow cytometry before being submitted to RNA sequencing. The results underline the role of mucosal hemocytes in peripheral immunity in marine bivalves.

## 2 Material and methods

### 2.1 Oysters

Adult *C. virginica* oysters (n = 24, 90–100 mm in height) were sourced from Frank M. Flowers and Sons Inc. (Oyster Bay, New York, USA) in August 2020. Upon arrival, the oysters were thoroughly cleaned to remove epibionts and debris. They were then acclimated to two 30-litre aquaria (salinity: 25 ppt; temperature: 20 °C) for two weeks, during which they were fed daily with a commercial diet (LPB Frozen Shellfish Diet^®^, Campbell, California, USA). Three days prior to the cell sorting, the oyster shells were carefully notched without damaging the underlying tissue. The oysters were then individually placed in 800 ml tanks containing filtered (1µm) seawater (salinity: 25 ppt; temperature: 20 °C), and water was changed daily.

### 2.2 Mucosal and circulatory hemocytes collection

Mucosal hemocytes were collected using a method adapted from Lau et al. (50), following an initial drainage of water from the pallial cavity to remove any debris. To facilitate hemocyte dissociation from the pallial surfaces, three ml of isosmotic ethylenediaminetetraacetic acid (EDTA) solution (1 L distilled water, 2.6 g NaH_2_PO_4_·H_2_O, 14.4 g Na_2_HPO_4_·2H_2_O, 10 g EDTA, 4 g NaCl; pH 7.4) (52) were pipetted into the notched area of the shell. Each oyster was carefully wrapped in Parafilm to prevent leakage and placed on a shaker for gentle swirling over a 30-minute period. The fluid was then pipetted out and examined under a microscope to confirm an adequate hemocyte count and absence of contaminants. Preliminary experiments indicated that draining residual water from the pallial cavity before introducing the EDTA solution significantly improved the hemocyte yield in the final fluid collection.

Hemolymph was collected from the adductor muscle using a syringe with a 26-gauge needle and diluted in a 1:1 ratio with the EDTA solution. After collection, all samples were immediately placed on ice and kept there until flow cytometry sorting, which was performed within two to three hours after sampling.

### 2.3 Flow cytometry

Prior to flow cytometry sorting, all samples were filtered through a 35 μm nylon mesh screen to remove debris and large aggregates. The samples were then sorted using a FACSAria flow cytometer (BD Biosciences, San Jose, CA), with a sterile saline (0.9% Sodium Chloride Irrigation USP, Travenol Canada Inc., Ontario) as the sheath fluid. Regions were gated and adjusted based on forward and side scatter signals to isolate and retrieve 40,000 agranulocytes and granulocytes from each body fluid sample. Sorted hemocytes were collected directly into 200 μl of lysis buffer from the RNAqueous Micro Kit (Ambion, AM1931). The cell lysate samples were kept on ice until RNA extraction, which was performed either on the same day or the following one. Representative aliquots of sorted hemocytes were also collected on microscope slides for visual verification of sample quality.

### 2.4 RNA isolation, library preparation, and sequencing

Total RNA from sorted cells of five oysters was extracted according to the RNAqueous Kit manufacturer’s protocol, with the goal of obtaining five replicates for each hemocyte subpopulation. RNA quality and quantity assessment, library preparation and sequencing were carried out at Genome Quebec (McGill University, Canada). The quantity and quality of RNA were assessed using a NanoDrop Spectrophotometer ND-1000 (NanoDrop Technologies, Inc.). RNA integrity was verified using a 2100 Bioanalyzer (Agilent Technologies). Three out of the 20 hemocyte RNA extracts failed quality control, resulting in 17 samples that proceeded to sequencing. For cDNA synthesis, 30 ng of total RNA per sample was processed using the NEBNext^®^ Single Cell/Low Input RNA Library Prep Kit for Illumina^®^ (New England BioLabs). Library quantification was performed with the Kapa Illumina GA with Revised Primers-SYBR Fast Universal kit (Kapa Biosystems), and fragment sizes were checked with a LabChip GX (PerkinElmer) instrument. Libraries were normalized, pooled, denatured in 0.05N NaOH, and neutralized with HTI buffer. The pool was then loaded at 225 pM onto an Illumina NovaSeq S4 lane using Xp with a paired-end 2x100 cycle format, as per the manufacturer’s instructions. A PhiX library was included as a 1% control. Base calling was performed with RTA v3.4.4 software, and raw sequence data in FASTQ format were generated by demultiplexing the sample using bcl2fastq2 v2.20 software.

### 2.5 RNA-seq data processing and analysis

Raw FASTQ files were processed using the nf-core RNA-seq pipeline (nf-core/rnaseq v3.14.0) (54–56). Adapter sequences and low-quality bases were trimmed with Trim Galore v0.6.7 (57) and Cutadapt v3.4 (58). The cleaned reads were then aligned to the eastern oyster reference genome (GCA_002022765.4, C_virginica-3.0) (59) using the STAR v2.7.9a (60) and quantified with Salmon v1.10.1 (61). PCA was performed on variance-stabilized expression values after removing oyster ID effects using the removeBatchEffect() function in the limma package v3.60.6, with tissue origin and cell type included in the design matrix to reduce inter-individual effects in the ordination. Associations of PC scores with tissue origin, cell type, and library size were tested using Type II ANOVA. The quantified transcripts were analyzed using the nf-core/differentialabundance pipeline (nf-core/differentialabundance v1.4.0) (54, 55, 62), which performs differential expression testing in DESeq2 (v1.34.0) (63) while accounting for inter-individual variation by including oyster ID as a blocking factor in the model. Genes were considered differentially expressed if they exhibited a Benjamini–Hochberg adjusted p-value (false discovery rate, FDR) < 0.05 and an absolute log_2_ fold change > 1. Heatmaps were generated using ClustVis software (https://biit.cs.ut.ee/clustvis/) (64). Gene Ontology (GO) molecular function (MF), biological process (BP), and cellular component (CC) (65) and Kyoto Encyclopedia of Genes and Genomes (KEGG) (66) pathway enrichment analyses were conducted using g:Profiler (g:GOSt) (67) with multiple-testing correction based on the g:SCS method. To avoid bias introduced by non-expressed genes, the background universe was restricted to the 38,719 genes retained after low-abundance filtering in the nf-core RNA-seq workflow (i.e., genes tested for differential expression). To explicitly account for gene-length–related detectability bias in RNA-seq enrichment analyses, GO enrichment was additionally evaluated using goseq v1.50.0 (Wallenius method) (68), with gene lengths derived from the reference gene annotation.

## 3 Results

### 3.1 Flow cytometry results

**Figure 1:**
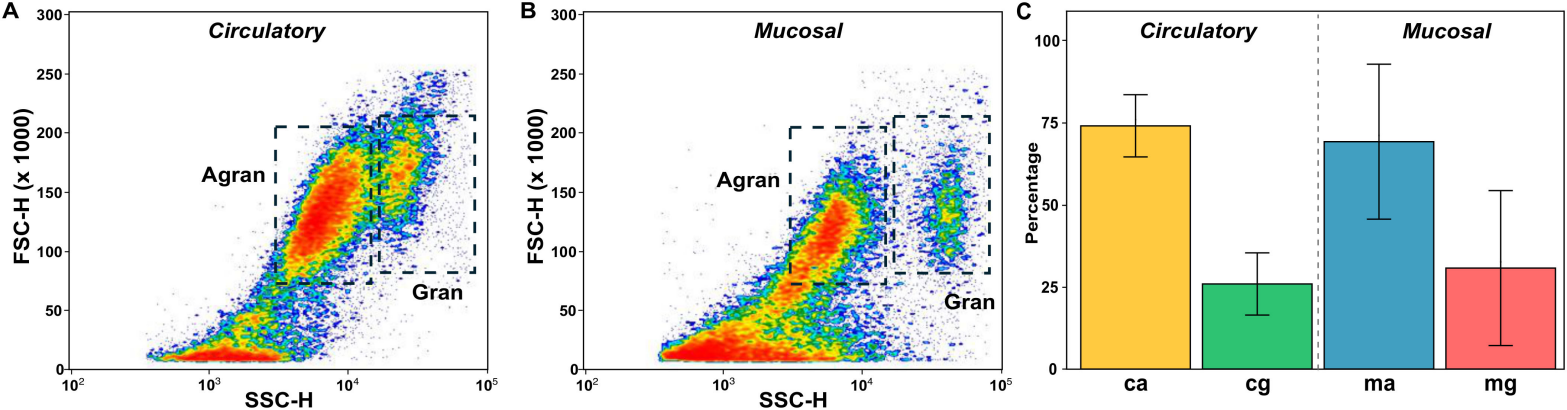
Density plots representing circulatory **(A)** and mucosal **(B)** hemocytes based on side (SSC) and forward (FSC) scatter measurements. Gates used to sort granulocytes (Gran) and agranulocytes (Agran) are shown. **(C)** Mean  ±  SD  percentages of granulocytes and agranulocytes from five oysters used for sorting and RNA extraction. Abbreviations: ca (circulatory agranulocyte), cg (circulatory granulocyte), ma (mucosal agranulocyte), mg (mucosal granulocyte).

Granulocytes and agranulocytes subpopulations were distinguishable in both hemolymph and pallial mucus (**Figure 1**), with granulocytes displaying higher side scatter than agranulocytes.

Both sample types displayed significantly higher percentage of agranulocytes (69.3% ± 23.6% and 74.1% ± 9.5%, mean ± SD for mucosal and circulatory samples, respectively) as compared to granulocytes (p<0.05, Student’s t-test), although no significant differences in hemocyte composition were noted between both body fluids.

### 3.2 RNA-seq data processing and exploratory analysis

Some sorted hemocyte populations did not yield high quality RNA or did not produce good libraries and were not sequenced. Therefore, RNA sequencing was effectively performed on 17 hemocyte samples representing granulocytes and agranulocytes isolated from pallial mucus and hemolymph. Paired-end Illumina sequencing generated between 28.5 million and 129.4 million raw reads per sample, with an average of 76.4 ± 29.6 million reads (**Supplementary Table 1**). The raw transcriptome data have been deposited in the NCBI Sequence Read Archive (SRA) under accession number of SRP585325. After adapter and quality trimming, >99.9% of reads were retained, indicating high-quality input RNA and efficient library preparation. Reads were aligned to the *C. virginica* reference genome using STAR. On average, 69.6 ± 2.4% of the trimmed reads mapped uniquely to the genome, while 12.3 ± 0.8% aligned to multiple loci and 0.09 ± 0.02% mapped to too many loci to be confidently placed. Approximately 17.7 ± 3.0% of reads remained unmapped due to insufficient length after trimming, and an additional 0.2 ± 0.05% were not mapped due to other causes. These metrics demonstrate consistent sequencing depth and alignment quality across all samples. To explore the structure of transcriptional variation among sorted hemocytes, PCA was performed on normalized expression data.

**Figure 2:**
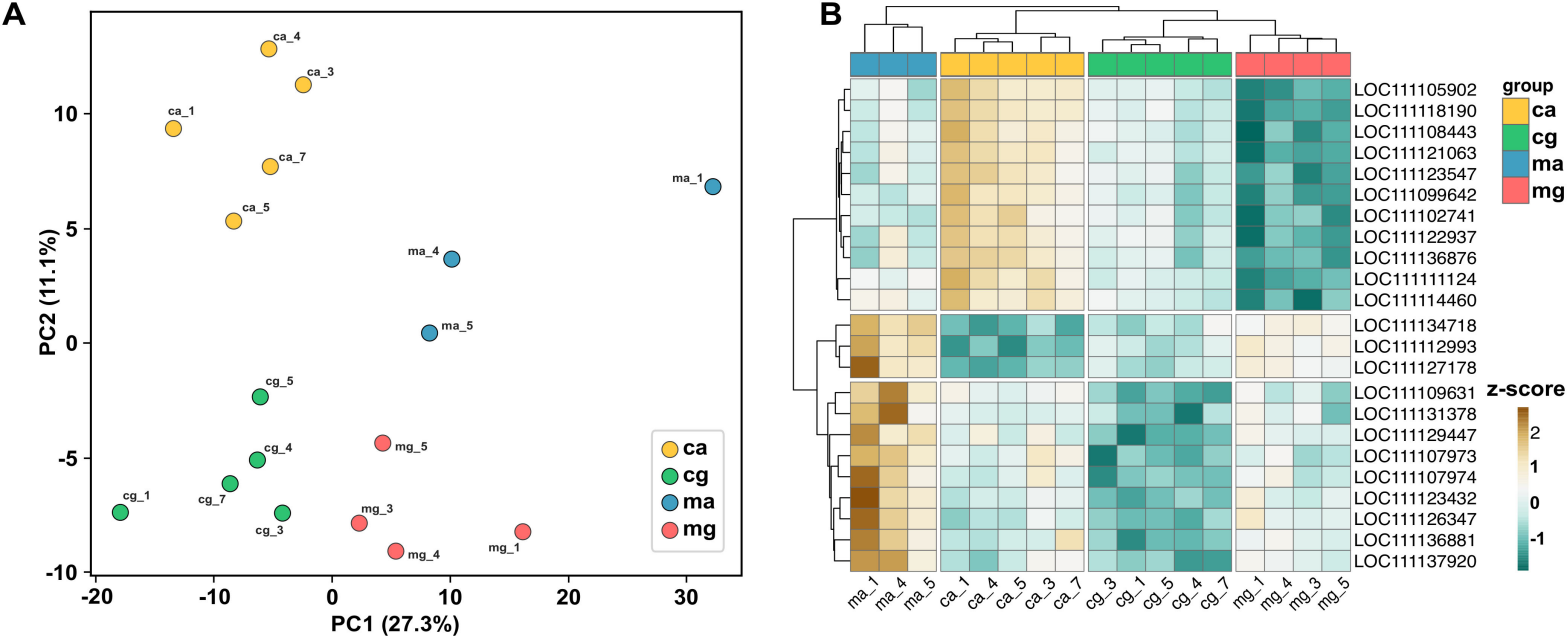
Transcriptomic structure of hemocyte populations. **(A)** PCA plot of gene expression data from *C. virginica* hemocyte subpopulations. Each point represents the transcriptomic profile of a biological replicate. **(B)** Heatmap of the relative expression levels across samples. Samples from different groups are shown in different colors. Expression data were normalized using variance stabilizing transformation; rows were scaled by z-score for visualization. The color of each cell indicates the scaled expression level of a gene, with a gradient ranging from teal (low expression) to brown (high expression). Genes shown in the heatmap were selected as a representative subset exhibiting clear expression differences among the four hemocyte groups. ma, mucosal agranulocyte; mg, mucosal granulocyte; ca, circulatory agranulocyte; cg, circulatory granulocyte. The numbers following the underscore represent the oyster number.

The resulting PCA plot revealed four clearly separated clusters corresponding to mucosal granulocytes, mucosal agranulocytes, circulatory granulocytes, and circulatory agranulocytes (**Figure 2A**). The first principal component primarily captured differences between mucosal and circulatory hemocytes, while the second component reflected variation between granulocytes and agranulocytes. This interpretation was supported by ANOVA of PC scores: tissue origin was significantly associated with PC1 (F = 25.8, p = 2.11×10^−4^) and cell type with PC2 (F = 95.9, p = 2.29×10^−7^), while library size was not associated with either axis (p > 0.5). Consistent differences in gene expression among mucosal and circulatory hemocytes, as well as between granulocytes and agranulocytes, were also apparent at the level of individual transcripts (**Figure 2B**). To characterize these transcriptional differences in greater detail, differential gene expression analysis was performed through pairwise comparisons between the hemocyte groups.

### 3.3 DEGs

**Figure 3:**
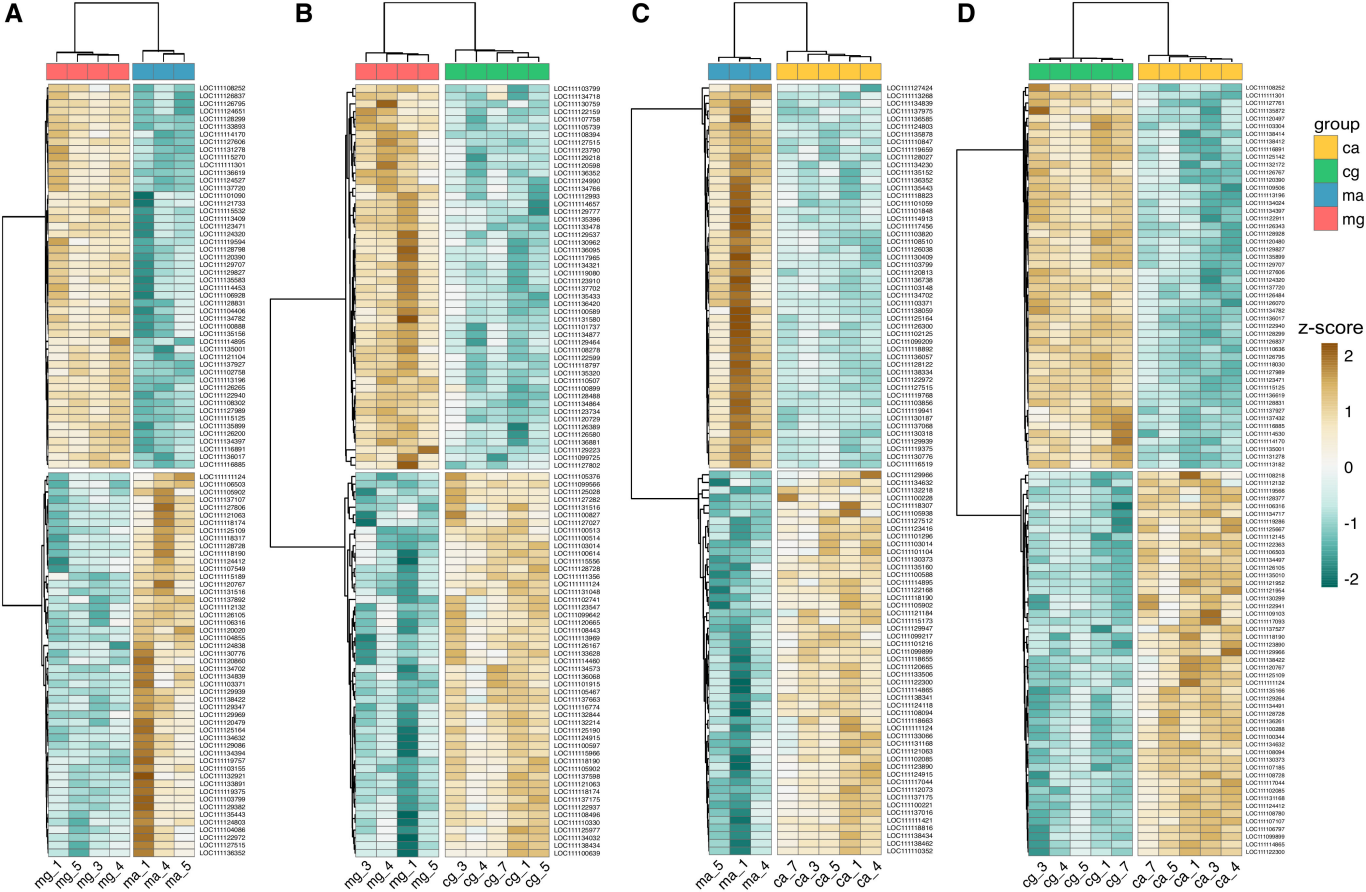
Expression profiles of differentially expressed genes across hemocyte groups. **(A)** Mucosal agranulocytes and mucosal granulocytes. **(B)** Circulatory and mucosal granulocytes. **(C)** Circulatory and mucosal agranulocytes. **(D)** Circulatory agranulocytes and circulatory granulocytes. Each panel shows a heatmap of the 50 genes with positive and the 50 with negative log_2_ fold change (ranked by adjusted *p*-value) from the corresponding comparison. Expression data were normalized using variance stabilizing transformation; rows were scaled by z-score for visualization. The color gradient ranges from teal (low expression) to brown (high expression). Sample groups are indicated by color bars. Numbers following the underscore represent individual oyster IDs. ma, mucosal agranulocyte; mg, mucosal granulocyte; ca, circulatory agranulocyte; cg, circulatory granulocyte.

Pairwise differential expression analysis was conducted between the four hemocyte subpopulations to identify genes with significant transcriptional differences. Replication was reduced for some groups due to RNA QC failures, which may reduce power for those comparisons. The highest number of differentially expressed genes (3,625) was detected between circulatory and mucosal agranulocytes. Comparisons between circulatory agranulocytes and granulocytes, and between circulatory and mucosal granulocytes, yielded 1,892 and 1,630 DEGs, respectively. The lowest number (888) was observed between mucosal agranulocytes and granulocytes. Expression profiles of the top 50 DEGs revealed consistent transcriptomic divergence between all hemocyte subpopulations, with clear clustering by both cell type and tissue origin (**Figure 3**).

**Figure 4:**
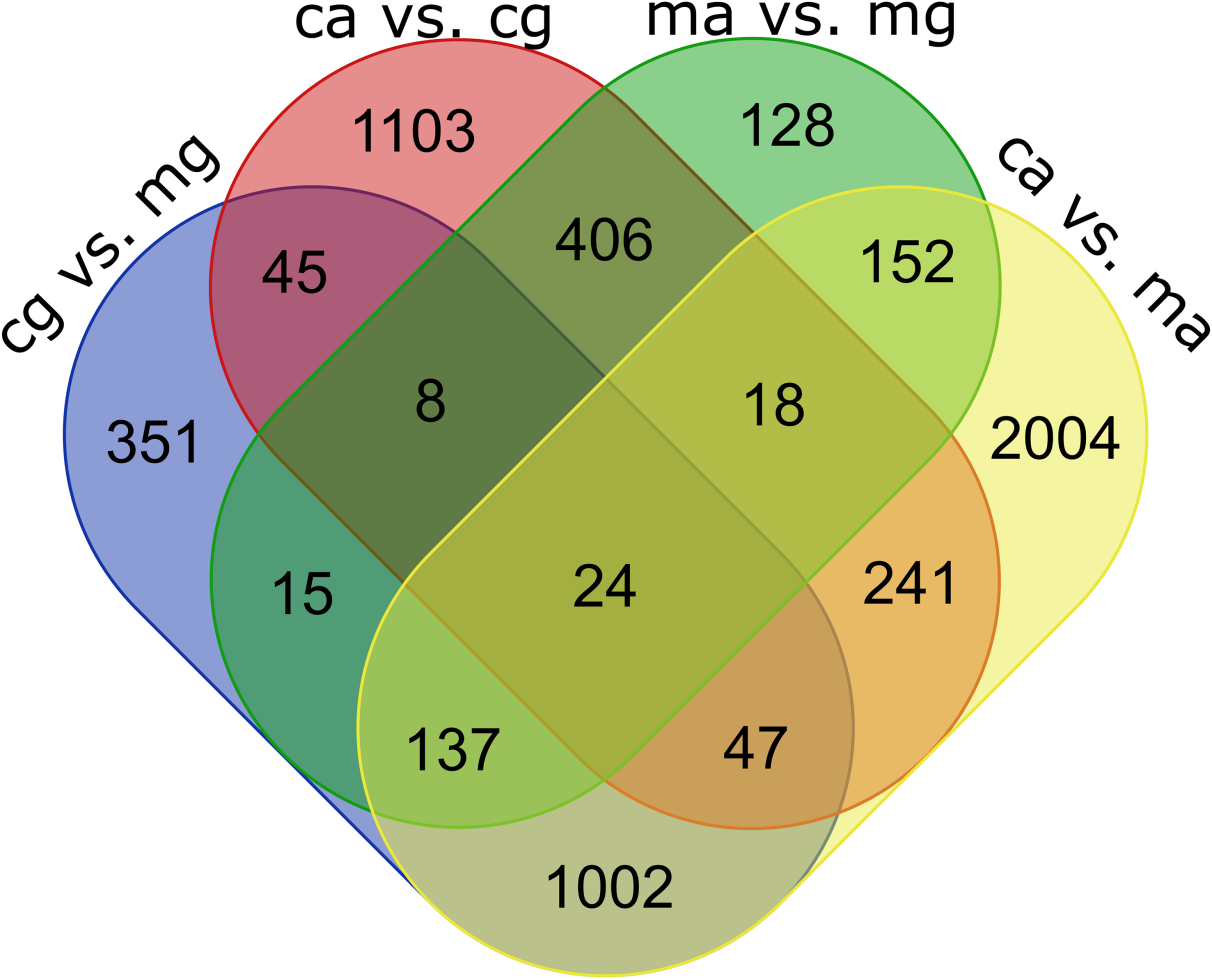
Venn diagram illustrating the number of common and unique DEGs across hemocyte subpopulations. ma, mucosal agranulocyte; mg, mucosal granulocyte; ca, circulatory agranulocyte; cg, circulatory granulocyte.

Some genes were differentially expressed in only one comparison, while others were shared across multiple groups. The extent of overlap is summarized in **Figure 4**, and the full DEG lists are provided in **Supplementary Tables 2**–**5**.

In addition to the individual comparisons, overlapping sets of differentially expressed genes were examined to identify expression patterns associated with either hemocyte origin or cell type. A total of 1,002 genes were found to be consistently differentially expressed between hemocytes originating from the circulatory system and those from the pallial mucus, both in granulocyte and agranulocyte populations. Similarly, 406 genes were shared between comparisons of granulocytes and agranulocytes across both origins, indicating expression differences primarily associated with cell type.

#### 3.3.1 Differential expression between circulatory and mucosal granulocytes

Comparison of gene expression profiles between granulocytes isolated from the pallial mucus and those derived from the hemolymph revealed a total of 1,630 DEGs (**Supplementary Table 2**). Of these, 1,354 transcripts exhibited higher abundance in mucosal granulocytes, while only 276 genes showed higher expression in circulatory granulocytes.

**Figure 5:**
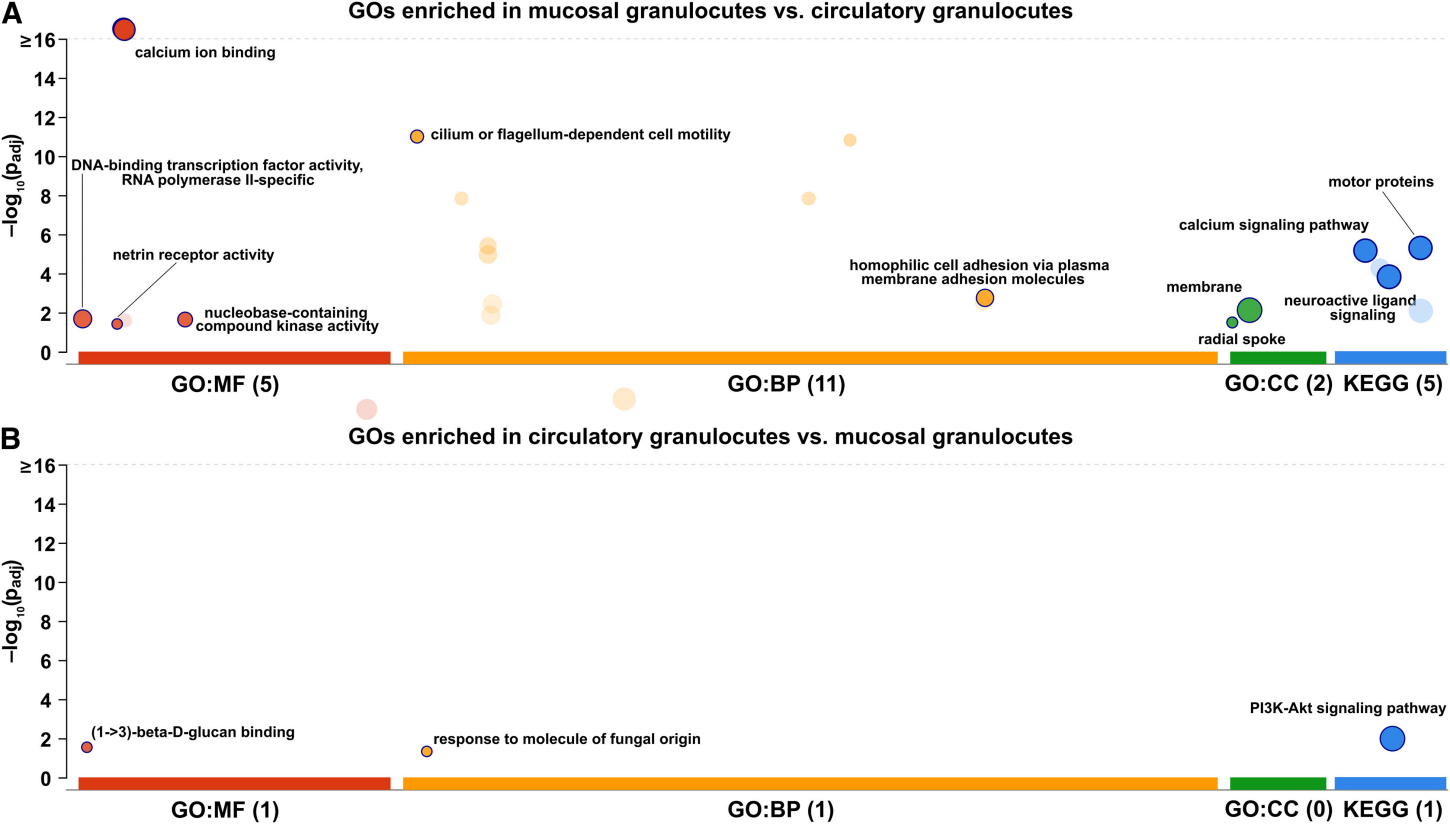
GO enrichment analysis of differentially expressed genes between circulatory and mucosal granulocytes. Circles indicate enriched GO terms (molecular function, MF; biological process, BP; cellular component, CC) and KEGG pathways for genes more highly expressed in **(A)** mucosal granulocytes and **(B)** circulatory granulocytes. The y-axis represents the adjusted p-values (–log_10_ scale), and circle size indicates the number of associated genes. Dark circles highlight the specific categories retained after semantic filtering by g:Profiler.

Genes more highly expressed in mucosal granulocytes were significantly enriched across 18 GO categories (**Supplementary Table 6**). Within the calcium ion binding (GO:0005509) category, 85 transcripts exhibited higher expression in mucosal granulocytes, highlighting significant differences in calcium-mediated processes compared to circulatory granulocytes (**Figure 5A**). This category encompassed multiple transcripts strongly associated with biomineralization and shell formation, including a large group of calcium-dependent regulatory proteins. Specifically, 13 calmodulin and calmodulin-like genes, 5 neo-calmodulin-like genes, caltractin-like (LOC111136013), and calbindin-32-like (LOC111136252) genes. In addition, the set of upregulated transcripts included 15 cadherin and protocadherin-like genes, which may contribute to cell adhesion and organization within the pallial cavity. Other transcripts within this functional group encompassed a diverse array of calcium-binding proteins, such as EF-hand domain–containing genes (e.g., LOC111119770, LOC111104008), and annexins (LOC111121260, LOC111121288, LOC111126580, LOC111121229).

Within the DNA-binding transcription factor activity, RNA polymerase II-specific (GO:0000981) category, 16 transcripts showed higher expression in mucosal granulocytes. The majority of these were homeobox-containing genes, including paired box protein Pax-6-like (LOC111104117, LOC111110798), homeobox protein six1-like (LOC111129404, LOC111129405), homeobox protein Dlx6a-like (LOC111125464), and engrailed-like genes (LOC111104532, LOC111101308). Additional upregulated transcripts included LIM homeobox genes (LOC111126347, LOC111136375), and POU domain transcription factors (LOC111127064, LOC111126745). Within the nucleobase-containing compound kinase activity (GO:0019205) category, mucosal granulocytes showed increased transcription of adenylate kinase (e.g., LOC111125074, LOC111130301), thymidine kinase, and thioredoxin domain-containing kinase genes. The netrin receptor activity (GO:0005042) category included transcripts encoding netrin receptor UNC5 homologs (LOC111131792, LOC111116639, LOC111126949).

Enriched BP term cilium or flagellum-dependent cell motility (GO:0001539) included radial spoke head proteins homologs (LOC111129218, LOC111129936), tektin-3-like (LOC111134864, LOC111120729), tektin-2-like (LOC111118797, LOC111099776), and cilia- and flagella-associated protein 46-like (LOC111134585).

KEGG enrichment analysis revealed five functional pathways among genes more highly expressed in circulatory granulocytes. The neuroactive ligand–receptor interaction (map04080) and glutamatergic synapse (map04724) pathways were consistent with the overrepresentation of G protein-coupled receptor activity and transporter activity identified in the GO analysis. Similarly, pyruvate metabolism (map00620) and mineral absorption (map04978) were associated with oxidoreductase activity, NAD binding, and membrane transport functions.

In contrast, only two GO terms were enriched among genes more highly expressed in circulatory granulocytes (**Figure 5B**). These were (1→3)-β-D-glucan binding (GO:0001872) and response to molecule of fungal origin (GO:0002238). Both categories were driven by the same pair of transcripts: LOC111108604 and LOC111108605, encoding perlucin-like protein sequences. KEGG enrichment analysis identified the PI3K-Akt signaling pathway (map04151) among genes more highly expressed in circulatory granulocytes compared to mucosal granulocytes. This group included multiple collagen genes (LOC111134682, LOC111132274), hepatocyte growth factor receptor-like transcripts (LOC111110330, LOC111108496), and matrilin-2-like (LOC111127282), all of which are associated with extracellular matrix organization and cell–matrix interactions. In addition, the pathway included toll-like receptor 6 (LOC111109802), a serine protease inhibitor Cvsi-2-like (LOC111126725), and a multiple epidermal growth factor-like domains protein (LOC111138434), representing components linked to immune signaling and regulatory processes.

#### 3.3.2 Differential expression between circulatory and mucosal agranulocytes

**Figure 6:**
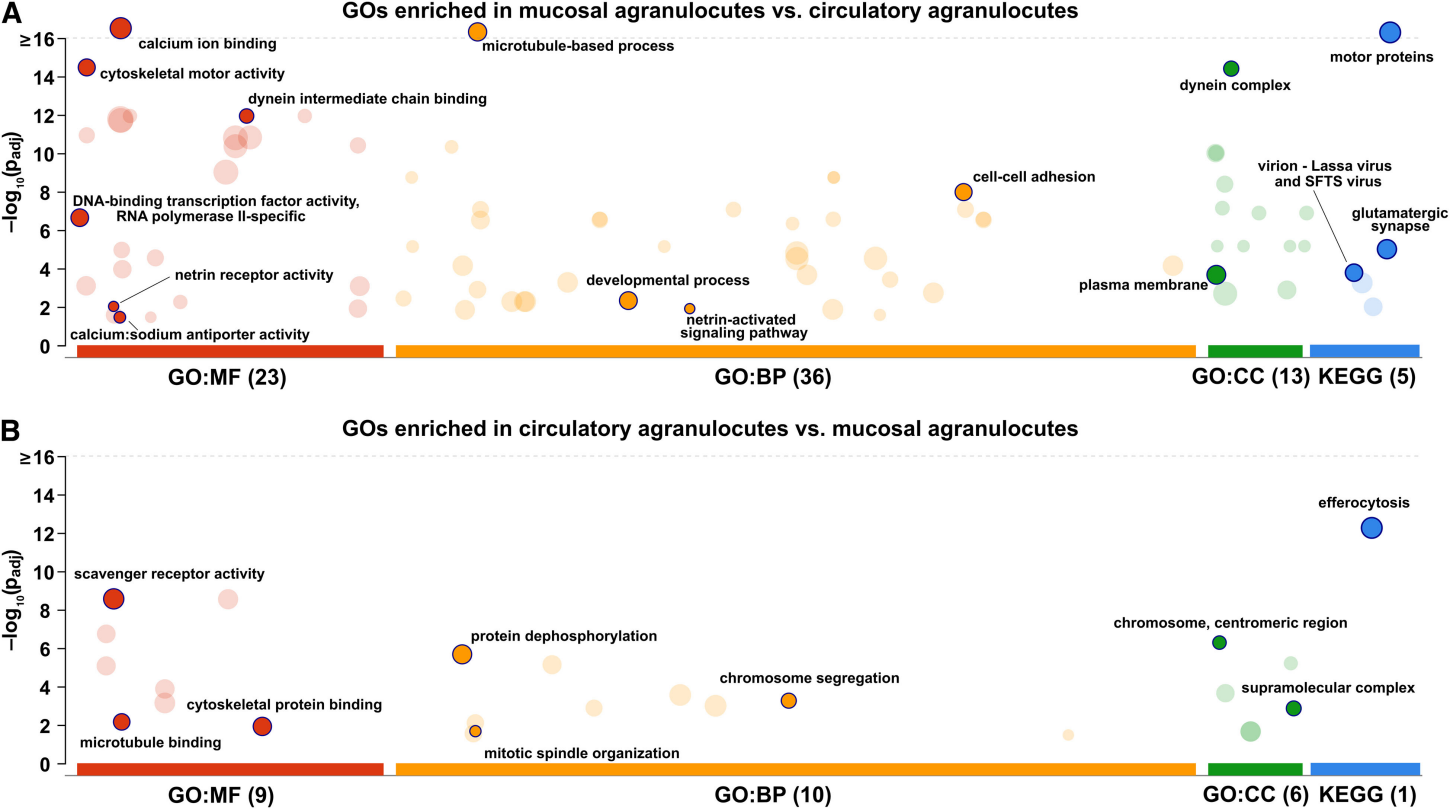
GO enrichment analysis of differentially expressed genes between circulatory and mucosal agranulocytes. Circles indicate enriched GO terms (molecular function, MF; biological process, BP; cellular component, CC) and KEGG pathways for genes more highly expressed in **(A)** mucosal agranulocytes and **(B)** circulatory agranulocytes. The y-axis represents the adjusted p-values (–log_10_ scale), and circle size indicates the number of associated genes. Dark circles highlight the specific categories retained after semantic filtering by g:Profiler.

Comparison of gene expression profiles between circulatory and mucosal agranulocytes revealed a total of 3,625 DEGs (**Supplementary Table 3**). Of these, 3,139 transcripts exhibited higher abundance in mucosal agranulocytes, while 486 genes were more highly expressed in their circulatory counterparts. Genes more highly expressed in mucosal agranulocytes were significantly enriched across 72 GO categories (**Figure 6A**; **Supplementary Table 7**). Within the calcium ion binding (GO:0005509) category, 186 transcripts exhibited higher expression in mucosal agranulocytes, representing the most significantly enriched functional group. This category included 23 calmodulin-like, 26 cadherin and protocadherin-like, 12 EF-hand domain-containing, 6 von Willebrand factor A domain-containing protein-like, 5 annexin-like, 5 fibrillin-like, and 2 fibropellin-like genes. This pattern of calcium ion binding gene enrichment closely resembles that observed in mucosal granulocytes, suggesting that overrepresentation of genes involved in calcium signaling, cellular regulation, and shell formation is a feature specific to the pallial compartment, regardless of hemocyte subtype. The cytoskeletal motor activity (GO:0003774) category comprised 41 dynein, myosin and kinesin family genes (e.g., LOC111134888, LOC111124554, LOC111127315).

Within the DNA-binding transcription factor activity, RNA polymerase II-specific (GO:0000981) category, 36 transcripts exhibited higher expression in mucosal agranulocytes. Most of these genes belonged to major homeobox families, including Hox (e.g., LOC111135933), Six (e.g., LOC111129405), Dlx (e.g., LOC111125464), and Engrailed (e.g., LOC111109706), alongside paired box (Pax-6-like; e.g., LOC111104117), and LIM homeobox transcription factors (LOC111136375). Within the netrin receptor activity (GO:0005042) category, four transcripts encoding netrin receptor homologs were upregulated in mucosal agranulocytes, including netrin receptor UNC5B-b-like (LOC111116639, LOC111131792) and UNC5C-like proteins (LOC111118753, LOC111126949). Transcripts annotated with calcium:sodium antiporter activity (GO:0005432) included sodium/calcium exchanger 1-, 2-, and 3-like genes (e.g., LOC111129095, LOC111129088, LOC111111628), indicating an enrichment of ion transporters involved in calcium and sodium homeostasis.

Within the microtubule-based process (GO:0007017) category, transcripts upregulated in mucosal agranulocytes included a broad range of genes encoding structural and regulatory components of the microtubule cytoskeleton. Notably, this group comprised numerous dynein heavy and beta chain genes, kinesin-like proteins (e.g., KIF4-like: LOC111130568; KIF9: LOC111127315, LOC111122805; KIF28P: LOC111108234, LOC111134572), tektins (e.g., tektin-3-like: LOC111120071, LOC111134864; tektin-1-like: LOC111135089), and radial spoke head protein homologs (e.g., LOC111129250, LOC111129936). Additional upregulated transcripts included tubulin alpha and beta chain genes (LOC111137486, LOC111112993) and centrosome-associated proteins (LOC111124172, LOC111122689), underscoring the functional specialization of mucosal agranulocytes for motility and microtubule-dependent intracellular transport.

KEGG enrichment analysis identified several pathways among genes more highly expressed in mucosal agranulocytes compared to circulatory agranulocytes. The motor proteins pathway (map04810) included transcripts encoding myosin (LOC111132357), dynein chains (e.g., LOC111130438, LOC111129377), and tctex1 proteins (LOC111119275, LOC111118129, LOC111118298). The glutamatergic synapse pathway (map04724) contained ionotropic glutamate receptor genes (LOC111104821, LOC111104802, LOC111136802) and excitatory amino acid transporters (LOC111103311, LOC111112640, LOC111132162, LOC111137550).

Among genes exhibiting higher expression in circulatory agranulocytes, enrichment was observed in 25 GO categories (**Figure 6B**). Within the scavenger receptor activity (GO:0005044) category, 32 transcripts were upregulated, predominantly represented by protein draper-like genes (27 transcripts, e.g., LOC111116590, LOC111137132, LOC111103511), multiple epidermal growth factor-like domain-containing proteins (3 transcripts, e.g., LOC111111417, LOC111099430), and scavenger receptor cysteine-rich domain-containing protein (LOC111111953). Within the protein tyrosine phosphatase activity (GO:0004725) and protein dephosphorylation (GO:0006470) categories, 20 transcripts exhibited higher expression. This group was dominated by receptor-type tyrosine-protein phosphatases, including alpha-like (LOC111113763), mu-like (LOC111112753), kappa-like (LOC111121063), eta-like (LOC111103943), and epsilon-like (LOC111116880), along with several uncharacterized and EGF-like domain–containing proteins. In the microtubule binding (GO:0008017) category, eight transcripts showed increased abundance, including centromere protein F-like (LOC111116905, LOC111128864), spindle and kinetochore-associated protein 1-like (LOC111130048), and several kinesin family members (e.g., LOC111107338, LOC111118975). The cytoskeletal protein binding (GO:0008092) category comprised 12 transcripts, notably myosin heavy chain, striated muscle-like (LOC111124621, LOC111120907), and filamin-A-like (LOC111119371). The chromosome segregation (GO:0007059) and chromosome, centromeric region (GO:0000775) categories each included seven transcripts, represented by kinetochore protein NDC80 homolog (LOC111126458), centromere protein F-like (LOC111116905, LOC111128864), and structural maintenance of chromosomes protein 4-like (LOC111120473). The mitotic spindle organization (GO:0007052) category contained three transcripts, including targeting protein for Xklp2 homolog (LOC111132092, LOC111132031).

KEGG enrichment analysis identified the efferocytosis pathway (map04148) among genes more highly expressed in circulatory agranulocytes compared to mucosal agranulocytes. This pathway was mainly represented by transcripts associated with scavenger receptor activity, cargo receptor activity, and protein dephosphorylation. In addition to these groups, the pathway included adhesion G protein-coupled receptor E1-like (LOC111126799), thrombospondin-1-like (LOC111138401), GTP-binding protein YPTC1-like (LOC111130783), several prostaglandin E2 receptor EP4 subtype-like transcripts (LOC111118663, LOC111118308, LOC111118307, LOC111118242), and neurogenic locus notch homolog protein 2-like (LOC111111415).

#### 3.3.3 Differential expression between mucosal granulocytes and agranulocytes

To characterize gene expression differences between hemocyte types originating from the pallial mucus, transcriptomic profiles of granulocytes and agranulocytes isolated from this site were compared. This analysis identified 888 differentially expressed genes (**Supplementary Table 4**). Of these, 475 genes were expressed at significantly higher levels in granulocytes, while 413 showed higher expression in agranulocytes.

**Figure 7:**
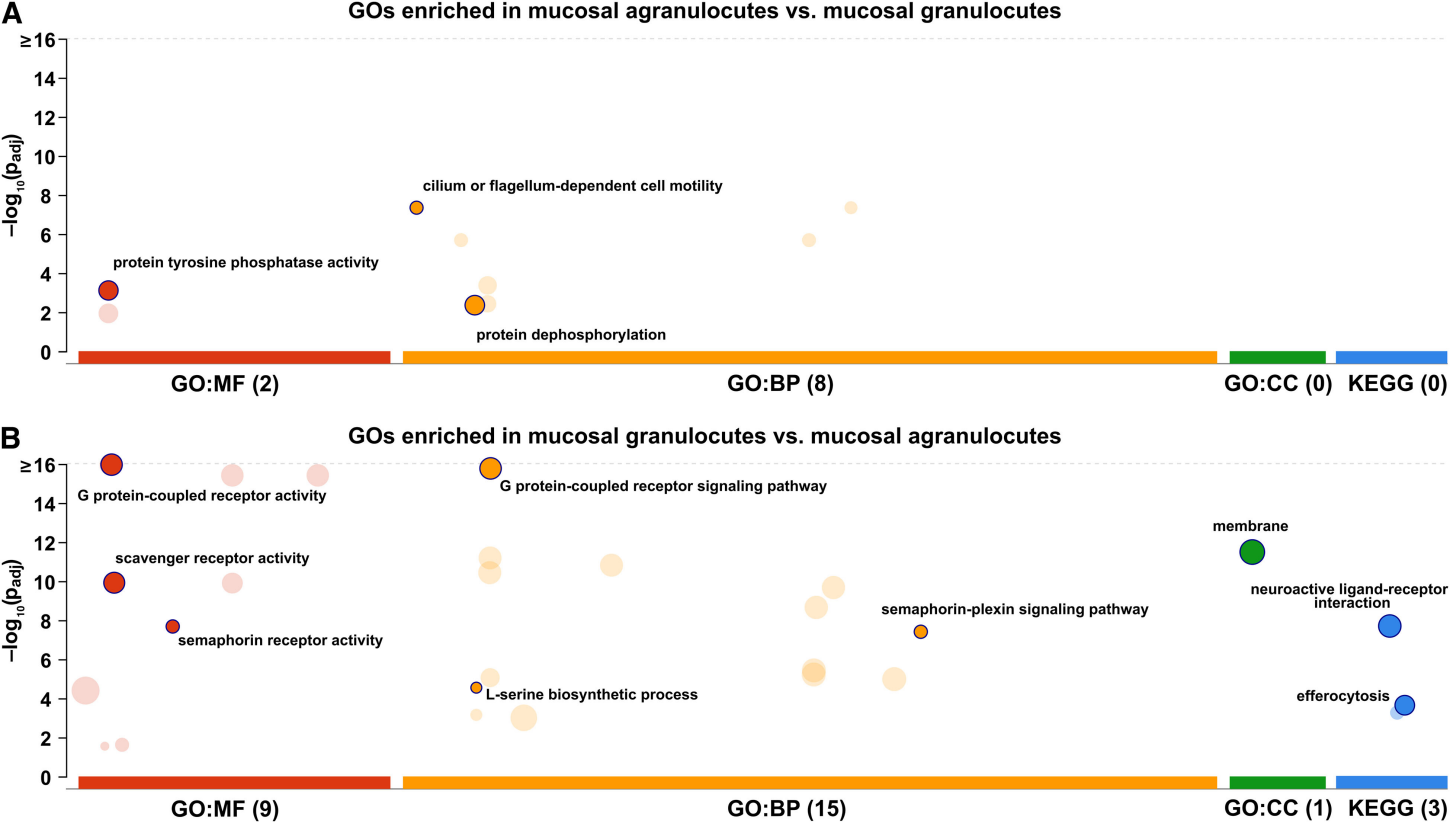
GO enrichment analysis of differentially expressed genes between mucosal hemocyte populations. Circles indicate enriched GO terms (molecular function, MF; biological process, BP; cellular component, CC) and KEGG pathways for genes more highly expressed in **(A)** mucosal agranulocytes and **(B)** mucosal granulocytes. The y-axis represents the adjusted p-values (–log_10_ scale), and circle size indicates the number of associated genes. Dark circles highlight the specific categories retained after semantic filtering by g:Profiler.

Genes that are more highly expressed in mucosal agranulocytes as compared to granulocytes were categorized using GO terms in both molecular function (MF), describing the biochemical activity of gene products, and biological process (BP) (**Supplementary Table 8**). Enriched MF terms included protein tyrosine phosphatase activity (GO:0004725) (**Figure 7A**). The transcripts annotated as receptor-type tyrosine-protein phosphatase T-like (LOC111110390, LOC111111948 and LOC111107549) were among these. Enriched BP terms included cilium or flagellum-dependent cell motility (GO:0001539) and protein dephosphorylation (GO:0006470). Genes associated with ciliary structure and movement included tektin-3-like (LOC111134864), nephrocystin-4-like (LOC111129936), and flagellar-associated protein 44-like (LOC111120729). The dephosphorylation category included the same group of tyrosine phosphatases listed above.

Genes more highly expressed in mucosal granulocytes were enriched for MF terms such as G protein-coupled receptor activity (GO:0004930), scavenger receptor activity (GO:0005044), semaphorin receptor activity (GO:0017154), O-phospho-L-serine:2-oxoglutarate aminotransferase activity (GO:0004648), and phosphoglycerate dehydrogenase activity (GO:0004617) (**Figure 7B**). These categories included several G protein-coupled receptors (GPCRs), such as cephalotocin receptor 1-like (LOC111116253), prokineticin receptor 2-like (LOC111120245), and cardioacceleratory peptide receptor-like (LOC111123471), as well as uncharacterized GPCR-like transcripts (LOC111115824, LOC111099818). Enzymes associated with amino acid biosynthesis and signal regulation included phosphoserine aminotransferase-like (LOC111115125), phosphoglycerate dehydrogenase-like (LOC111128299), and arrestin domain–containing protein 17-like (LOC111127989). BP terms enriched in the same group included G protein-coupled receptor signaling pathway (GO:0007186), semaphorin-plexin signaling pathway (GO:0071526), and L-serine biosynthetic process (GO:0006564). Consistently, KEGG enrichment analysis indicated that genes more highly expressed in mucosal granulocytes were enriched in the Neuroactive ligand–receptor interaction (map04080) pathway, which includes various GPCRs, as well as in the efferocytosis (map04148) pathway. In the KEGG context, the latter comprises genes associated with receptor-mediated phagocytic processes, and not exclusively with apoptotic cell clearance.

#### 3.3.4 Differential expression between circulatory granulocytes and agranulocytes

Comparative transcriptomic analysis of granulocytes and agranulocytes isolated from the hemolymph revealed 1,892 DEGs (**Supplementary Table 5**). Of these, 760 genes exhibited higher transcript levels in agranulocytes, while 1,132 genes were more abundantly expressed in granulocytes.

**Figure 8:**
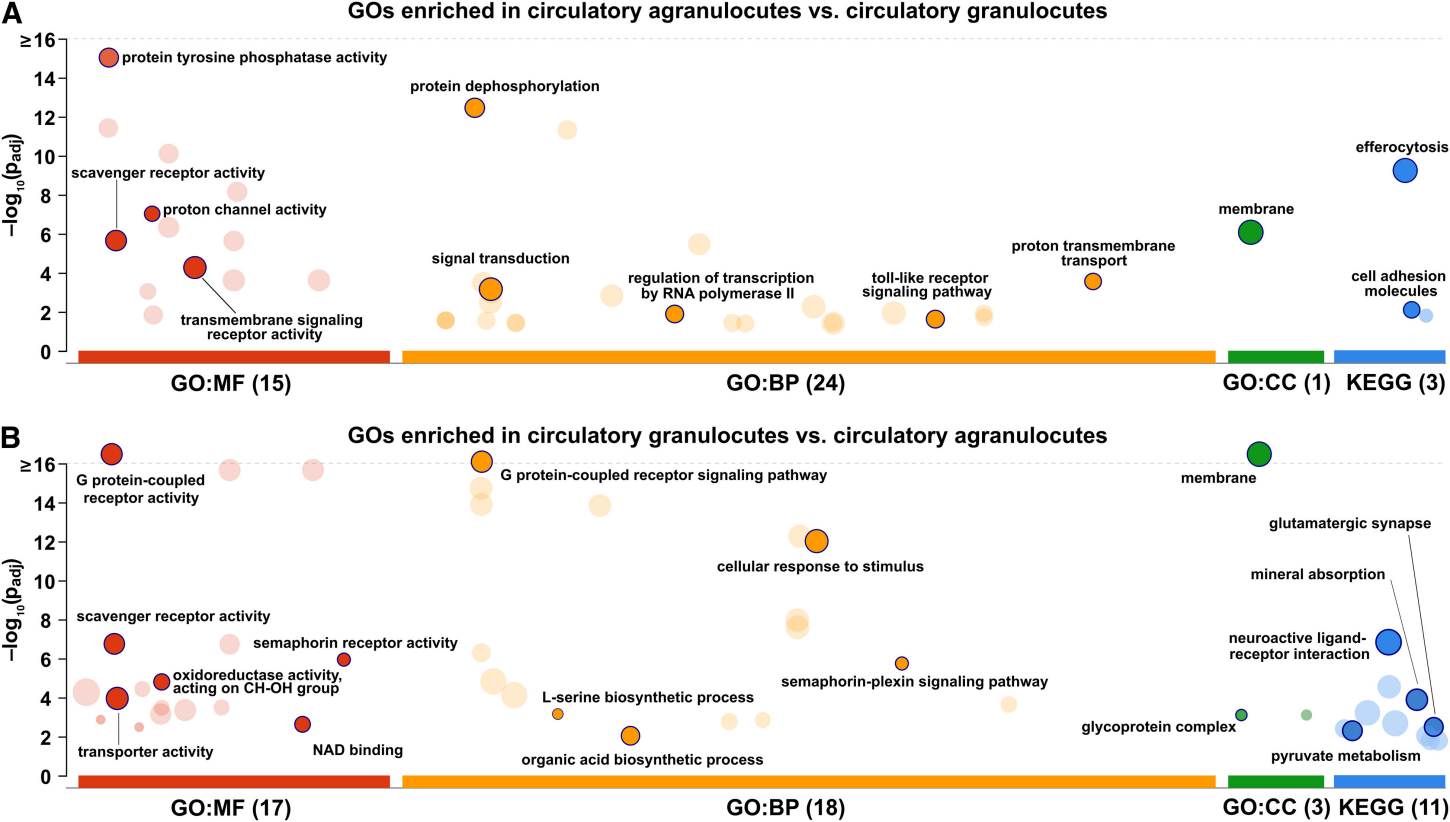
GO enrichment analysis of differentially expressed genes between circulatory hemocyte populations. Circles indicate enriched GO terms (molecular function, MF; biological process, BP; cellular component, CC) and KEGG pathways for genes more highly expressed in **(A)** circulatory agranulocytes and **(B)** circulatory granulocytes. The y-axis represents the adjusted p-values (–log_10_ scale), and circle size indicates the number of associated genes. Dark circles highlight the specific categories retained after semantic filtering by g:Profiler.

Within the MF category, transcripts expressed at significantly higher levels in agranulocytes than in granulocytes were strongly enriched for protein tyrosine phosphatase activity (GO:0004725) (**Figure 8A**), comprising 41 transcripts. Many of these encoded receptor-type tyrosine phosphatases, including kappa-like (LOC111113771, LOC111118174, LOC111121063), alpha-like (LOC111110653, LOC111113763), T-like (LOC111105863, LOC111109191), mu-like (LOC111112753, LOC111113743), and epsilon-like (LOC111112683, LOC111116880) (**Supplementary Table 9**). This set also included delta-, eta-, H-, F-, and C-like phosphatases, reflecting transcriptional activation of diverse signaling regulators in circulatory agranulocytes.

Transcripts showing higher expression in agranulocytes were also enriched for proton channel activity (GO:0015252), comprising 13 genes, including a predicted voltage-gated hydrogen channel 1-like gene (LOC111136839). Scavenger receptor activity (GO:0005044) included diverse immune-related transcripts, such as protein draper-like (LOC111113796, LOC111114400), scavenger receptor class F member 2-like (LOC111111471), and platelet endothelial aggregation receptor 1-like (LOC111115003), along with 29 multiple epidermal growth factor-like domain proteins. Transcripts annotated with transmembrane signaling receptor activity (GO:0004888) included a wide array of signaling molecules, such as 15 toll-like receptors, dopamine receptor 1-like (LOC111135860), prostaglandin E2 receptor EP4 subtype-like (e.g., LOC111118663), and several neuropeptide and hormone receptors, including sex peptide receptor-like (e.g., LOC111114261) and gonadotropin-releasing hormone receptor-like (LOC111128485). In the BP category, enriched terms included protein dephosphorylation (GO:0006470), proton transmembrane transport (GO:1902600), signal transduction (GO:0007165), regulation of transcription by RNA polymerase II (GO:0006357), and deoxyribonucleotide catabolic process (GO:0009264). Additionally, several GO terms related to immune activation were enriched, including toll-like receptor signaling pathway (GO:0002224).

Consistently, KEGG enrichment analysis showed that genes more highly expressed in circulatory agranulocytes were significantly enriched in the efferocytosis (map04148) and cell adhesion molecules (map04514) pathways. The efferocytosis pathway was shaped not only by scavenger receptors and signaling molecules identified in the GO analysis, but also included additional transcripts annotated as caspase-14-like (LOC111106653), thrombospondin-1-like (LOC111138401), and several multiple epidermal growth factor-like domain proteins (e.g., LOC111100313, LOC111115464, LOC111105266, LOC111113425). Enrichment of the cell adhesion molecules pathway reflected both receptor-type protein tyrosine phosphatases already discussed in the context of protein tyrosine phosphatase activity and protein dephosphorylation, as well as unique adhesion-related genes such as hemicentin-1-like (LOC111134632) and integrin beta-1-A-like (LOC111113326).

Genes more highly expressed in circulatory granulocytes exhibited a distinct set of enriched GO terms reflecting differences in signal transduction, metabolism, and membrane localization. The most significant molecular function category was G protein-coupled receptor activity (GO:0004930) (**Figure 8B**), which included a number of transcripts encoding annotated and uncharacterized GPCRs such as cephalotocin receptor 1-like (LOC111116253), prostaglandin E2 receptor EP4 subtype-like (LOC111120245), cardioacceleratory peptide receptor-like (LOC111123471), and cholecystokinin receptor-like (LOC111115824, LOC111099818). Additional receptor-associated functions included semaphorin receptor activity (GO:0017154), represented by several plexin-A2-like proteins (LOC111106664, LOC111106666, LOC111106670, LOC111108113, LOC111108818) and two plexin-A3-like proteins (LOC111106668, LOC111108820), and scavenger receptor activity (GO:0005044), which included epidermal growth factor-like domains proteins (e.g., LOC111113251, LOC111133099), protein draper-like (e.g., LOC111108070, LOC111132296), and platelet endothelial aggregation receptor 1-like (LOC111102982).

Metabolic and catalytic functions were also enriched in granulocytes, including oxidoreductase activity acting on the CH-OH group of donors (GO:0016614) and NAD binding (GO:0051287). These categories encompassed a diverse set of oxidoreductases and dehydrogenases, including NADP-dependent malic enzyme-like genes (LOC111103557, LOC111102676, LOC111102444), alcohol dehydrogenase [acceptor]-like (LOC111114530, LOC111104083), and glyoxylate reductase/hydroxypyruvate reductase-like sequences (LOC111137432, LOC111133131, LOC111132145). The GO term transporter activity (GO:0005215), encompassing a broad array of membrane-associated transport mechanisms, was also enriched in granulocytes. These included several calcium-related exchangers and ATPases. Multiple zinc ion transporters were enriched in granulocytes including zinc transporter ZIP14-like (LOC111100029), ZIP10-like (LOC111103306), and zinc transporter 2-like (LOC111103740).

In the biological process category, many of the same receptor genes contributed to enrichment of G protein-coupled receptor signaling pathway (GO:0007186), cellular response to stimulus (GO:0051716) and semaphorin-plexin signaling pathway (GO:0071526). Additional metabolic processes were also overrepresented, such as L-serine biosynthetic process (GO:0006564), organic acid biosynthetic process (GO:0016053), and alpha-amino acid metabolic process (GO:1901605), involving shared transcripts including phosphoserine aminotransferase-like (LOC111115125) and D-3-phosphoglycerate dehydrogenase-like. The cellular component ontology was dominated by the GO term “membrane” (GO:0016020), consistent with the prevalence of transmembrane receptor transcripts in this group.

KEGG enrichment analysis revealed eleven functional pathways among genes more highly expressed in circulatory granulocytes as compared to agranulocytes. The neuroactive ligand–receptor interaction (map04080) and glutamatergic synapse (map04724) pathways were consistent with the overrepresentation of G protein-coupled receptor activity and transporter activity identified in the GO analysis. Similarly, pyruvate metabolism (map00620) and mineral absorption (map04978) were associated with oxidoreductase activity, NAD binding, and membrane transport functions.

## 4 Discussion

Barrier epithelia are the first tissues to encounter seawater-borne microorganisms, toxins and physical stressors. In bivalve mollusks, these interfaces include the gill, mantle and body-wall surfaces, which are continuously bathed in a viscous pallial mucus layer. The field of classical studies of oyster immunity has historically centered on circulatory hemocytes. However, recent research has identified a novel compartment of specialized cells, termed mucosal hemocytes, which reside within or are attached to the mucus covering pallial epithelia. This compartment is in direct contact with the environment and its biological functions are only beginning to be explored.

Our previous phenotypic investigations revealed that mucosal hemocytes exhibit different phenotypic characteristics (clusters of differentiation) and basal activities (e.g., phagocytosis, reactive-oxygen production) when compared with their hematogenous counterparts (50). Secondly, *in vivo* imaging and tracer experiments have demonstrated bidirectional transepithelial migration: mucosal hemocytes have been observed to cross the mantle epithelium into underlying tissues, while circulatory cells can be seen to exit to repopulate the mucus layer (49). Further, mucosal hemocytes were observed to internalize the protozoan parasite *Perkinsus marinus* and translocate it across the pallial epithelium into internal tissues, implicating them in pathogenesis as well as defense (9). In a recent study by Rey Campos et al. (69), immune cells collected from the intervalvar cavity of marine bivalves resisted apoptosis when exposed to UV and acute infections, while also exhibiting molecular signatures of migration and stress resistance. Interestingly, hemocytes from these bivalves were detected in the surrounding water and were shown to enter other individuals without triggering allorecognition, suggesting a transferable, population-level immune defense mechanism (69).

Collectively, these observations imply that mucosal hemocytes may function as sentinels, surveying a microbe-rich frontier and transmitting information to systemic immunity. Despite these insights, the molecular underpinnings of hemocyte niche specialization have remained obscure. Previous studies relied on cytological stains, limited flow-cytometric markers or targeted gene assays (49, 50, 70). The present study addresses this knowledge gap by generating *de novo* transcriptomes for granulocytes and agranulocytes isolated from both the pallial mucus and the hemolymph of *C. virginica*. PCA of the 17 transcriptomic libraries segregated samples into four distinct clusters, with tissue origin (mucosal vs. circulatory) representing the primary axis of separation, and cell morphology (granulocyte vs. agranulocyte) contributing secondary variation. Pairwise contrasts revealed thousands of DEGs between subpopulations, suggesting compartment-associated transcriptional specialization. Altogether, these findings suggest functional heterogeneity and potential contrast in differentiation states among hemocytes from both compartments, while acknowledging that these interpretations are inferred from bulk RNA-seq data rather than direct single-cell resolution.

### 4.1 Mucosal hemocytes

The oyster’s pallial cavity presents a drastically different environment compared to the internal circulation, primarily due to its constant exposure to seawater-borne microbes, particulate matter, and environmental stressors (71, 72). The pallial mucus, which covers the mantle and gill surfaces, represents a frontline barrier where hemocytes are subjected to distinct challenges. In this study, transcriptomic comparisons reveal that hemocytes associated with the pallial mucus, particularly agranulocytes, undergo profound functional specialization, consistent with prior findings (49, 50). Notably, our data show that mucosal agranulocytes exhibit the greatest number of total and unique DEGs when compared to their circulatory counterparts. Furthermore, both mucosal granulocytes and agranulocytes display a convergent shift in gene expression, highlighting a shared transcriptional response to local environmental conditions. The expression of thousands of genes differs between mucosal and hemolymph hemocytes overall, emphasizing the extent of niche-specific adaptation within oyster immune cell populations.

**Figure 9:**
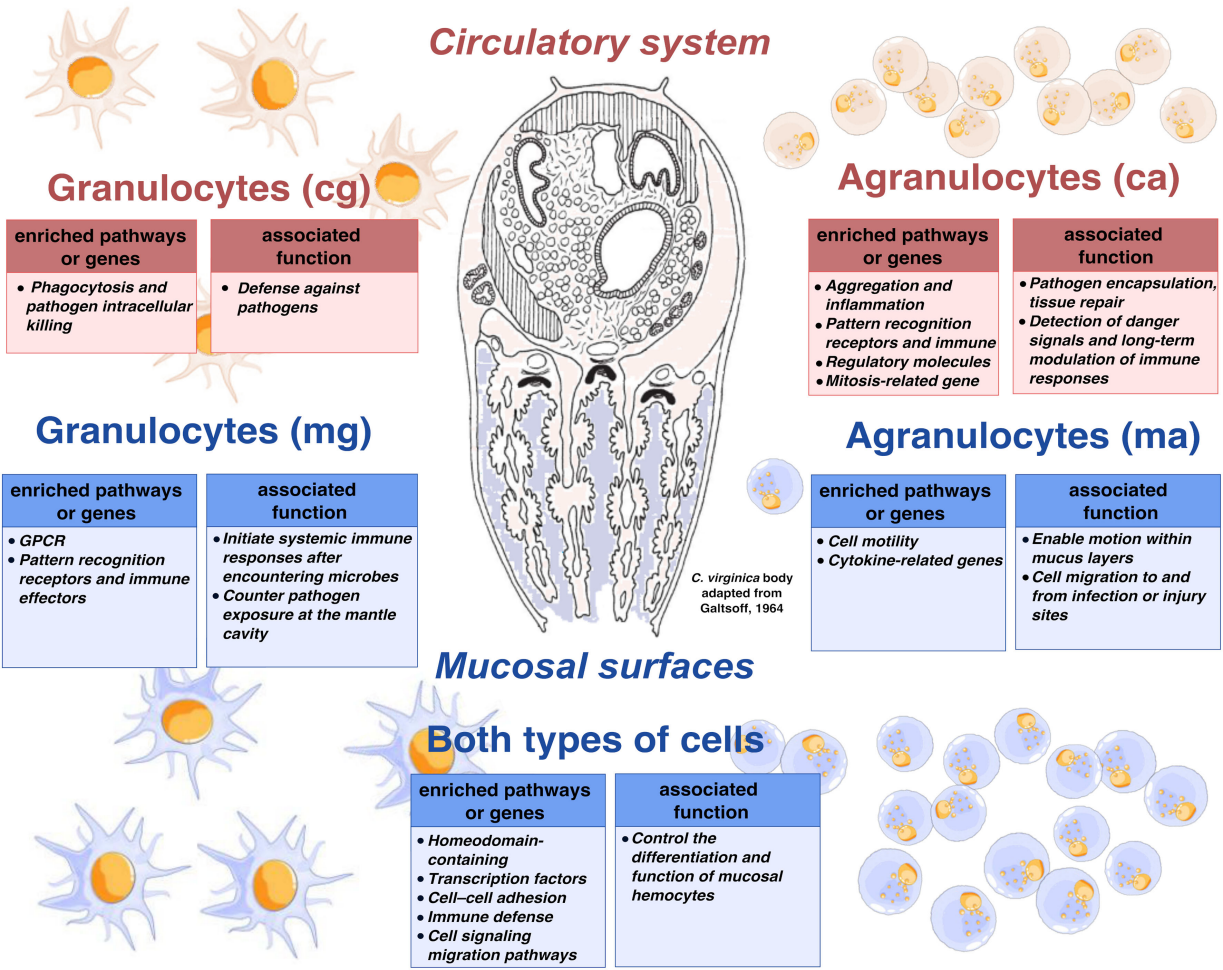
Proposed functional model highlighting transcriptional specializations of granulocytes and agranulocytes in pallial and circulatory compartments of *C. virginica*.

A dominant feature of this mucosal transcriptomic signature is the enrichment of functions related to calcium ion binding and cytoskeletal organization (**Figure 9**). Numerous transcripts encoding calcium-binding proteins are upregulated in both types of mucosal hemocytes. For instance, multiple calmodulin-like genes, EF-hand domain proteins, annexins, and some shell matrix-related proteins (e.g., fibropellins), which are well known for their roles in calcium signaling and biomineralization (73–76), show elevated expression in mucosal hemocytes, suggesting potential involvement in these processes. Their overrepresentation in the pallial compartment may indicate that hemocytes at the mantle interface may partake in shell maintenance by regulating or supplying ions and matrix components for calcification. This idea is supported by similar observations in other bivalves – extrapallial hemocytes (located between the mantle and shell) of bivalves upregulate a “biomineralization toolkit” of chitinases, carbonic anhydrases, and calcium-binding proteins while carrying higher intracellular Ca^2+^ loads (11, 13, 77). Notably, some of these calcium-binding proteins (e.g., C1q-domain proteins, certain annexins) have dual functions in immunity and biomineralization, hinting that mucosal hemocytes might be adapted to enable immune defense while potentially also contributing to shell formation (13). To confirm these findings, intracellular Ca^2+^ levels measurements using dyes or other complementary activity based assays (e.g., exchange between pallial and extrapallial hemocytes) would help validate their potential role in calcification.

Another shared transcriptional program in mucosal hemocytes involves cytoskeletal motor and microtubule-related genes. Increased expression of transcripts associated with cell motility was noted in mucosal hemocytes, particularly mucosal agranulocytes. The GO category “cilium or flagellum-dependent cell motility” was notably enriched, driven by elevated expression of dynein heavy chains, kinesin-family motors, radial spoke proteins, tektins, EF-hand domain-containing family proteins, RIB43A-like coiled-coil proteins, and multiple flagellar-associated proteins. EF-hand domain-containing proteins are known regulators of calcium-dependent signaling pathways crucial for cytoskeletal remodeling and motility (78), while RIB43A-like proteins stabilize microtubule structures and facilitate cell motility (79). Tektins and flagellar-associated proteins are fundamental structural and functional components essential for motility and microtubule stability in both ciliated and non-ciliated cells (80–82). These proteins may enable agranulocytes to actively propel themselves within mucus layers, physically sweep away foreign particles, or extend receptor-rich sensory appendages (e.g., filipodia) for microbial detection, substantially enhancing barrier immunosurveillance at the pallial surfaces. While direct ultrastructural confirmation remains necessary, the robust and coherent transcriptional signature identified here provides compelling support for this functional hypothesis. In vertebrates, dendritic cells migrate from peripheral tissues exposed to microbes to lymphoid tissues to illicit appropriate immune responses (83). In *C. virginica*, bidirectional transepithelial migration between circulatory system and mucosal surfaces was observed (50), and mucosal hemocytes have also been shown to transport the protozoan parasite *P. marinus* across pallial surfaces through transepithelial migration (9). Together with those previous findings, the DEGs, enriched KEGG pathways and GO terms identified here provide insights into the molecular genes that could be involved in bidirectional migration capability of mucosal hemocytes, suggesting they may migrate more effectively than their circulatory counterparts, functioning in a manner similar to dendritic cells (84, 85).

Alongside their enhanced motility capabilities, mucosal hemocytes exhibited significant enrichment of transcripts involved in cell signaling, adhesion, and guided migration pathways compared to their circulatory counterparts. Mucosal agranulocytes exhibited significantly elevated expression of eleven cytokine-related genes, including interleukin-17, bone morphogenetic proteins, growth/differentiation factors, and Wnt proteins. Cytokines play a crucial role in activating and regulating immune responses, inflammation, and hematopoiesis (86). Interleukin-17, a pro-inflammatory protein, regulates the NF-κB pathway triggering systemic immune responses in invertebrates, including oysters (87–89). Additionally, bone morphogenetic proteins and growth/differentiation factors, part of the transforming growth factor-beta (TGF- β) superfamily, regulate cell differentiation, growth, and inflammatory responses (90). The overexpressed Wnt proteins, which are glycoproteins, are known to be involved in regulating cell differentiation, proliferation, and migration (91). Cytokines mediate hemocyte recruitment enhancing cell-cell and cell-matrix interactions, supporting cell migration to and from infection or injury sites (92). Additionally, mucosal granulocytes showed elevated expression of multiple GPCR transcripts. GPCRs broadly function as transmembrane sensors of extracellular cues and are known to regulate immune cell activation, chemotaxis, and intercellular communication (93–95). The observed enrichment of GPCR-related transcripts in the pallial compartment may reflect the need for heightened environmental responsiveness in mucosal hemocytes, which operate at the interface with seawater and microbial stimuli. These receptors could enable granulocytes to detect locally released peptides, neuromodulators, or stress signals within the mantle mucus, thereby supporting rapid immune activation and directed positioning within the mucus.

Overall, these transcriptional distinctions in cytokine and GPCR-related signaling suggest mucosal hemocytes possess specialized molecular machinery that could enhance their capability for targeted recruitment and signaling within the mantle’s mucosal immune compartment. Furthermore, these findings are consistent with the hypothesis that mucosal hemocytes might help initiate systemic immune responses after encountering microbes akin to dendritic cells and neutrophils in vertebrates. Targeted validation of genes involved in cytokine signaling and cell migration, using approaches such as qPCR in combination with assays specifically evaluating the regulation of cytokines in mucosal (and circulatory) hemocytes following pathogen exposure, would help further evaluate our transcriptomic findings and support the potential sentinel role of mucosal hemocytes in oyster immunity. From an immunological perspective, mucosal granulocytes also displayed upregulation in a variety of pattern recognition receptors and immune effectors relative to circulatory granulocytes. For instance, transcripts encoding galectins (galectin-4-like), known to bind microbial glycans (96–98), and lysosomal enzymes such as lysozyme 3, a bacteriolytic enzyme that cleaves peptidoglycan in bacterial cell walls (99), are elevated in mucosal granulocytes. These suggest an enhanced microbicidal arsenal in mucosal granulocytes, presumably to counter the higher pathogen exposure at the mantle cavity.

Transcripts encoding developmental and guidance factors, particularly DNA-binding TFs of the homeobox superfamily, are strikingly upregulated in mucosal hemocytes. Both the granulocyte and agranulocyte subsets of mucosal hemocytes exhibit significant enrichment of homeodomain-containing transcription factors, including representatives of the Pax (e.g., Pax6-like), Six, Dlx, Engrailed, LIM-homeobox (e.g., Lhx9) and POU domain families. In oysters and other mollusks, homeobox transcription factors are classically involved in processes such as epithelial patterning, directed cell migration, tissue renewal and regulated apoptosis (100, 101). For example, the LIM-homeobox family, particularly ChLhx9, plays a crucial role in hemocyte renewal and immune function in *Magallana hongkongensis* oysters (101). The pronounced overexpression of these factors in mucosal hemocytes may reflect the need for positional sensing and adaptive interaction with epithelial and microbial signals, demands that are less acute for hemocytes circulating in the hemolymph. In addition, both mucosal granulocytes and agranulocytes increased the expression of transcripts encoding netrin receptors (UNC5-like), which are proteins involved in axon guidance (102). Netrin signaling could mediate the directed migration or adhesion of hemocytes within the complex pallial mucus matrix (103). This coordinated enrichment indicates that morphogenetic cues and adhesion pathways together govern hemocyte positioning, tissue anchoring, and functional specialization at the mantle interface. For instance, the upregulation of Wnt family members and neuroglian in mucosal hemocytes suggests that they may interact closely with the epithelium or the extracellular matrix (104–106). The adhesion and signaling cues probably enable mucosal hemocytes to home to the mucosal surface, form stable contacts and potentially modulate local tissue repair or barrier integrity. This interpretation is supported by the differential expression of genes involved in cell–cell and cell–matrix adhesion across mucosal hemocyte types. Mucosal granulocytes overexpress numerous cadherin and protocadherin transcripts, which may mediate adhesion between hemocytes and the extracellular matrix of the pallial tissues, or among hemocytes. Mucosal agranulocytes exhibit significant enrichment of gene ontology categories associated with cell–cell adhesion and homophilic interactions via plasma membrane adhesion molecules. They also upregulate a number of mucin-like and hemicentin-like genes. While mucins are gel-forming components that structure and protect the mucus barrier (107), hemicentin is a large extracellular matrix protein that is involved in cell anchorage and the maintenance of tissue cohesion (108). The expression of this protein suggests that mucosal hemocytes may stabilize their position at the epithelial surface or contribute to extracellular matrix remodeling in pallial tissues. Overall, these adhesion and guidance features support the idea that mucosal hemocytes may act as resident sentinels or scaffolding cells at the pallial surfaces, integrating with both epithelial tissue and mucus to maintain barrier integrity and localized immune defense.

Despite sharing the mantle mucus niche, granulocytes and agranulocytes remain transcriptionally and functionally distinct, each reflecting their characteristic roles in immune defense and tissue maintenance. Notably, the principal differences distinguishing these cell types in the pallial compartment are largely preserved in the circulatory system. Thus, the canonical division of labor between granulocytes and agranulocytes is maintained across tissue contexts, and the following section focuses on their functional specialization within the hemolymph.

### 4.2 Circulatory hemocytes

Among the unique properties distinguishing circulatory hemocytes from their mucosal counterparts, the most prominent is the pronounced enrichment of mitosis-related gene expression in circulatory agranulocytes. Hematopoiesis plays a crucial role in maintaining immunity by producing hemocytes. Our analysis showed that circulatory hemocytes, particularly agranulocytes exhibit a higher association with GO terms and gene clusters related to mitosis more than other subpopulations. The heightened mitotic activity in circulatory hemocytes aligns with prior observations of hematopoiesis in bivalves. Although hemocytes are broadly thought to originate from the connective tissue, mitotic activity has also been observed in hemocytes after their release into the hemolymph (109). For instance, mitotic activity has been reported in circulating hemocytes, specifically hemoblasts, in the clam *Tapes philippinarum* (110). Furthermore, Tirapé et al. (111) localized the Cg-tal (Tal1/SCL) transcription factor to the gastrula-trochophore stage during development in *Magallana gigas*, suggesting that mitotic divisions possibly occur within circulatory hemocytes, and recent studies reported the presence of blast-like cells in that species (27, 28). These findings suggest that circulatory hemocytes, particularly circulatory agranulocytes appear to be more mitotically active and could potentially represent a younger population than mucosal hemocytes. However, functional assay assessing cell-cycle progression (e.g., EdU incorporation or Ki-67 expression) are required to draw definitive conclusions regarding the differential positioning of *C. virginica* granulocytes and agranulocytes in hemocytes life cycle.

GO terms related to hemocyte aggregation and inflammation were significantly enriched in circulatory agranulocytes compared to their mucosal counterparts (**Figure 8B**). Hemocyte aggregation plays a crucial role in wound healing and pathogen containment, as demonstrated in clams like *Tridacna crocea* (112) and *Saccostrea glomerata* (113). These aggregation responses, along with encapsulation processes mediated by agranulocytes, suggest their potential role in isolating pathogens and tissue repair (114).

Similarly, GO terms associated with phagocytosis and pathogen intracellular killing were significantly enriched in circulatory granulocytes as compared to mucosal granulocytes. Granulocytes primarily perform phagocytosis, distinguishing them from agranulocytes in *Magallana gigas* (115), *Pinctada imbricata* (23), and *Ruditapes philippinarum* (116). Their heightened phagocytic activity in oysters (32, 91, 117) is likely linked to their capacity for intracellular pathogen destruction, as granulocytes contain granules filled with enzymes and reactive oxygen species capable of neutralizing foreign organisms (8).

The data obtained from the circulatory hemocytes of *C. virginica* demonstrate a distinct transcriptional dichotomy between granulocytes and agranulocytes, which mirrors the conventional division of labor observed in bivalve immunity. Circulatory granulocytes up-regulate a broad suite of genes associated with oxidative metabolism and phagocytic activity, including higher expression of key metabolic enzymes and pathways such as multiple NAD-dependent dehydrogenases, malic enzyme, and pyruvate metabolism genes, consistent with their role as professional effector cells. This is presumably in order to support robust reactive oxygen species production during the course of a pathogenic challenge (25, 118). These cells also exhibit an extensive repertoire of GPCRs and guidance molecules (notably semaphorin and plexin receptors) relative to agranulocytes (119, 120). Such a profile is consistent with a cell type poised to sense chemotactic cues, migrate rapidly to sites of infection, and mount potent antimicrobial responses (5, 121). Previous studies in the Pacific oyster *Magallana gigas* have similarly characterized granulocytes as the main immunocompetent hemocytes responsible for phagocytosis and oxidative burst (122, 123). Our transcriptomic findings support this paradigm at the molecular level, reinforcing the view that circulatory granulocytes are the oyster’s analogues of vertebrate neutrophils – short-lived, highly active phagocytes equipped for frontline defense (124).

By contrast, circulatory agranulocytes display a transcriptional program enriched for pattern recognition receptors and immune regulatory molecules (114). The analysis found that agranulocytes expressed significantly higher levels of at least 15 distinct Toll-like receptors, dozens of Draper-family scavenger receptors, and more than 40 receptor-type protein tyrosine phosphatases involved in signal modulation (125, 126). This array of receptors and regulators indicates that agranulocytes are equipped for sustained detection of danger signals and long-term modulation of immune responses (8). The elevated receptor-type protein tyrosine phosphatases and other signaling factors suggest a role in tuning inflammatory pathways and facilitating receptor-mediated phagocytic processes, including efferocytosis, rather than directly killing pathogens (127).

Our results reinforce that the granulocyte–agranulocyte dichotomy is deeply conserved within oyster hemolymph, reflecting distinct but complementary immune strategies. Granulocytes provide a frontline defense characterized by rapid microbial clearance through oxidative bursts and chemotactic responses, whereas agranulocytes predominantly fulfill regulatory roles, mediating debris clearance, immune modulation, and tissue homeostasis. This fundamental division aligns closely with prior cytological studies in bivalves and parallels the vertebrate innate immune model of neutrophils and macrophages.

## 5 Conclusion

This study advances our understanding of mucosal immunity in marine bivalves by characterizing hemocyte subpopulations associated with pallial mucus and hemolymph in *C. virginica*. By integrating flow cytometry with transcriptomic analysis, we proposed distinct functional roles for mucosal agranulocytes and granulocytes, which suggest responsiveness to the unique environmental challenges encountered at the pallial surface. Gene expression profiles of mucosal hemocytes reveal elevated cell motility, signaling, cytokine activity, cell adhesion, and calcium ion binding, consistent with roles in transepithelial migration, microbial sampling, and initiation of immune responses. These attributes support the hypothesis that mucosal hemocytes may serve a role analogous to dendritic cells in vertebrates. On the other hand, circulatory agranulocytes exhibited transcriptomic signatures associated with mitosis, inflammation and aggregation, while circulatory granulocytes showed enrichment in pathways involved in phagocytosis and pathogen destruction compared to their mucosal counterparts. Altogether, these findings support the existence of an “externalized immune defense system” in bivalves as recently suggested by Rey Campos et al. (69). Future research should prioritize single-cell sequencing to determine whether mucosal hemocytes represent a specialized subpopulation of circulatory hemocytes and improve our knowledge regarding hematopoiesis in bivalves. Microbial challenge experiments and functional assays are also required to further characterize the functional role of mucosal hemocytes, particularly in sampling microbes and eliciting systemic immune responses.

## Data Availability

The datasets presented in this study can be found in online repositories. The names of the repository/repositories and accession number(s) can be found in the article/**Supplementary Material**.
